# Supplementary material for: Retrotransposon instability dominates the acquired mutation landscape of mouse induced pluripotent stem cells
Source: Nat Commun. 2022 Dec 3;13:7470. doi: 10.1038/s41467-022-35180-x (PMC9719517; doi:10.1038/s41467-022-35180-x)
Supplement: Supplementary file 1 — Supplementary Information [file 41467_2022_35180_MOESM1_ESM.pdf]

Supplementary Information

Retrotransposon instability dominates the acquired mutation landscape of mouse induced pluripotent stem cells

Gerdes P. et al.

10 Supplementary Figures

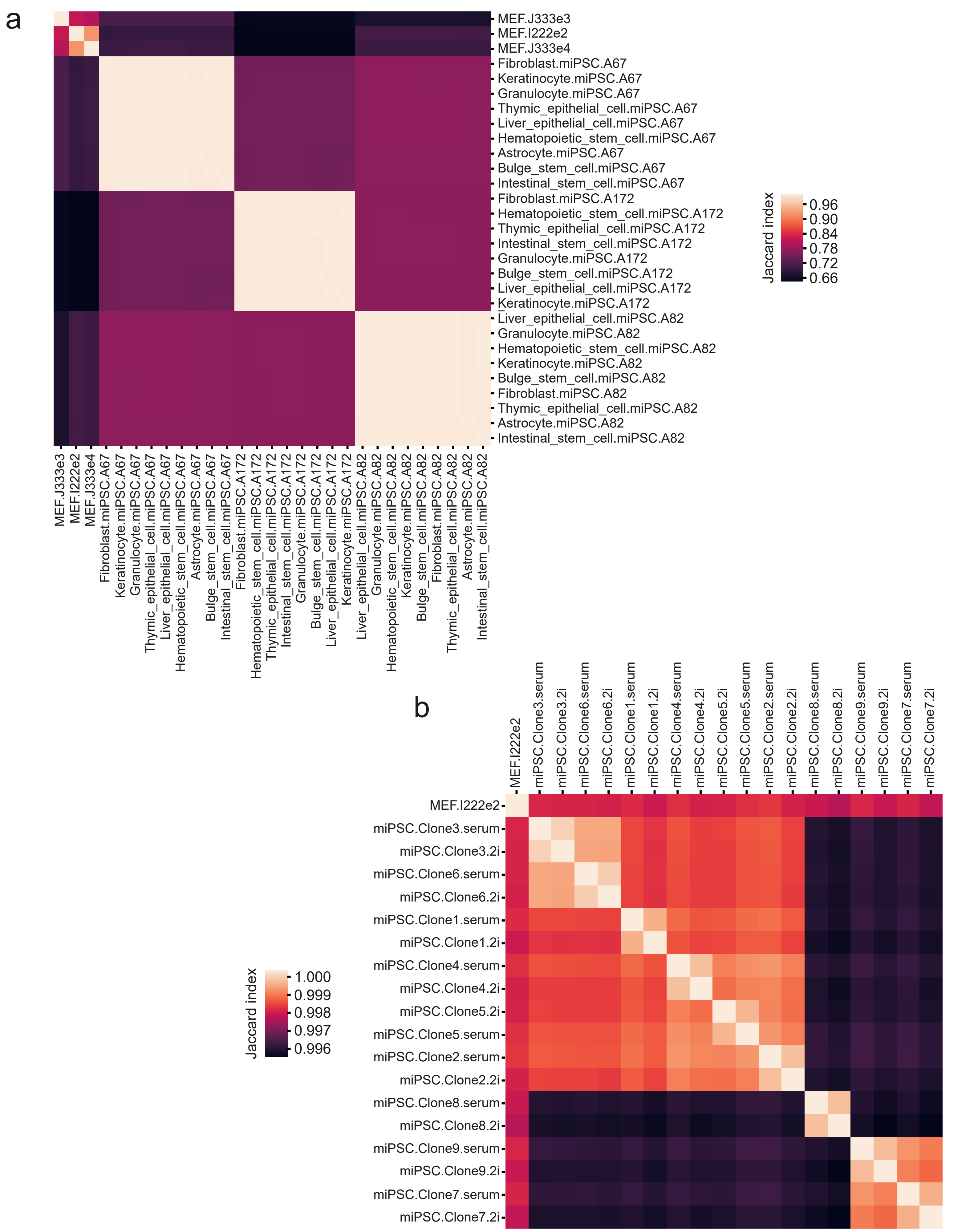

Supplementary Fig. 1. miPSC line genotypic relationships. a, Clustering of miPSC lines derived from 9 primary cell types isolated from 3 animals (A67, A82, A172), and 3 MEF genotypic controls. For each pairwise comparison, the Jaccard index (J) was calculated as the ratio of the union and intersection of SNP/INDEL variants called from WGS data and shared by the sample pair. Known SNPs/INDELs were removed and filtered as described in the Methods. J=1 (light color on key) indicates an identical variant profile between a sample pair, whereas J=0 (dark color on key) indicates no variants in common. Hierarchical clustering was performed using average linkage and a Euclidean distance metric via the seaborn clustermap function. **b,** As for panel (a), except for 9 single-cell clones derived from animal I222e2 MEFs and cultured in serum or 2i conditions.

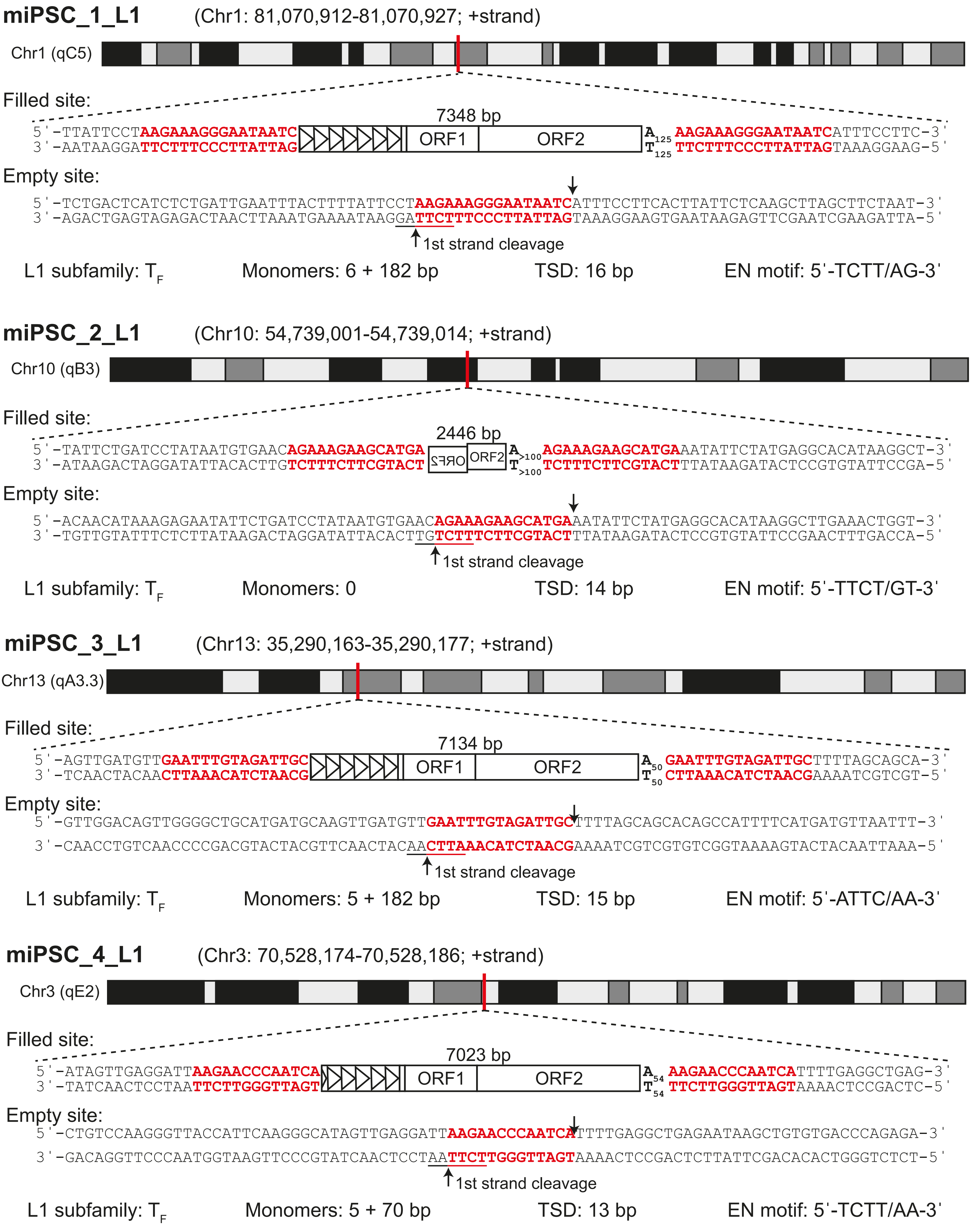

Supplementary Fig. 2. Sequence characteristics of de novo L1 insertions detected in bulk tissue-derived miPSCs. For each of four insertions, the following information is provided: the chromosomal location; a filled site illustration indicating target site duplication (TSD) sequences in red, the number of promoter monomers (black triangles) if applicable, and 3′ polyA tract length (A_n/T_n); an empty site illustration depicting TSD sequence and first strand endonuclease (EN) cleavage motif (underlined); summary characteristics (L1 subfamily, number of monomers, TSD length and EN motif).

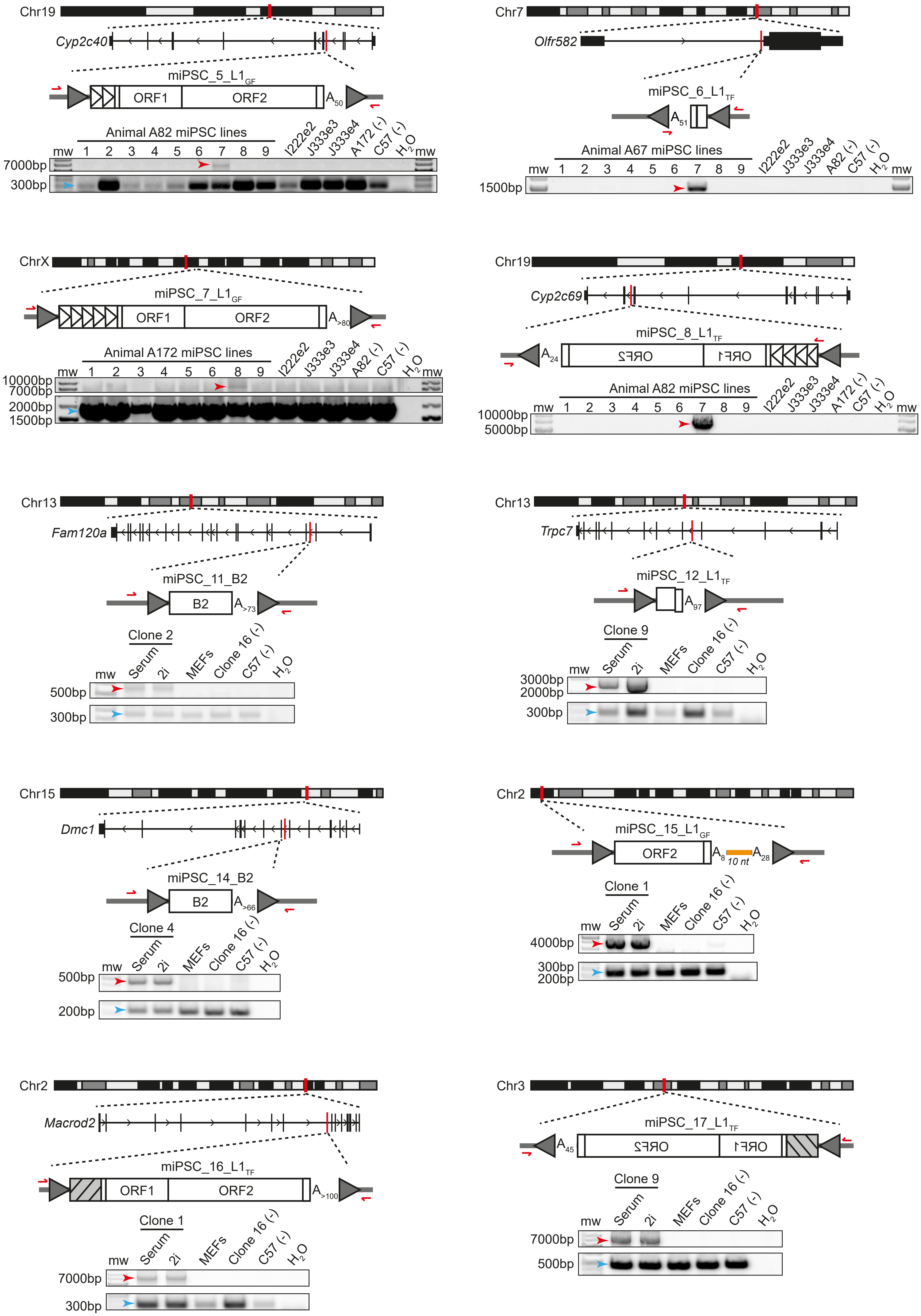
Chr19
Cyp2c40
miPSC_5_L1GF
ORF1
ORF2
A50
Animal A82 miPSC lines
mw 1 2 3 4 5 6 7 8 9
I222e2
J333e3
J333e4
A172 (-)
C57 (-)
H2O
mw
7000bp
300bp
Chr7
Olfr582
miPSC_6_L1TF
A51
Animal A67 miPSC lines
mw 1 2 3 4 5 6 7 8 9
I222e2
J333e3
J333e4
A82 (-)
C57 (-)
H2O
mw
1500bp
ChrX
miPSC_7_L1GF
ORF1
ORF2
A>80
Animal A172 miPSC lines
mw 1 2 3 4 5 6 8 9
I222e2
J333e3
J333e4
A82 (-)
C57 (-)
H2O
mw
10000bp
7000bp
2000bp
1500bp
Chr19
Cyp2c69
miPSC_8_L1TF
A24
ORF2
ORF1
Animal A82 miPSC lines
mw 1 2 3 4 5 6 7 8 9
I222e2
J333e3
J333e4
A172 (-)
C57 (-)
H2O
mw
10000bp
5000bp
Chr13
Fam120a
miPSC_11_B2
B2
A>73
Clone 2
mw
Serum
2i
MEFs
Clone 16 (-)
C57 (-)
H2O
500bp
300bp
Chr13
Trpc7
miPSC_12_L1TF
A97
Clone 9
mw
Serum
2i
MEFs
Clone 16 (-)
C57 (-)
H2O
3000bp
2000bp
300bp
Chr15
Dmc1
miPSC_14_B2
B2
A>66
Clone 4
mw
Serum
2i
MEFs
Clone 16 (-)
C57 (-)
H2O
500bp
200bp
Chr2
miPSC_15_L1GF
ORF2
A8
10 nt
A28
Clone 1
mw
Serum
2i
MEFs
Clone 16 (-)
C57 (-)
H2O
4000bp
300bp
200bp
Chr2
Macrod2
miPSC_16_L1TF
ORF1
ORF2
A>100
Clone 1
mw
Serum
2i
MEFs
Clone 16 (-)
C57 (-)
H2O
7000bp
300bp
Chr3
miPSC_17_L1TF
A45
ORF2
ORF1
Clone 9
mw
Serum
2i
MEFs
Clone 16 (-)
C57 (-)
H2O
7000bp
500bp

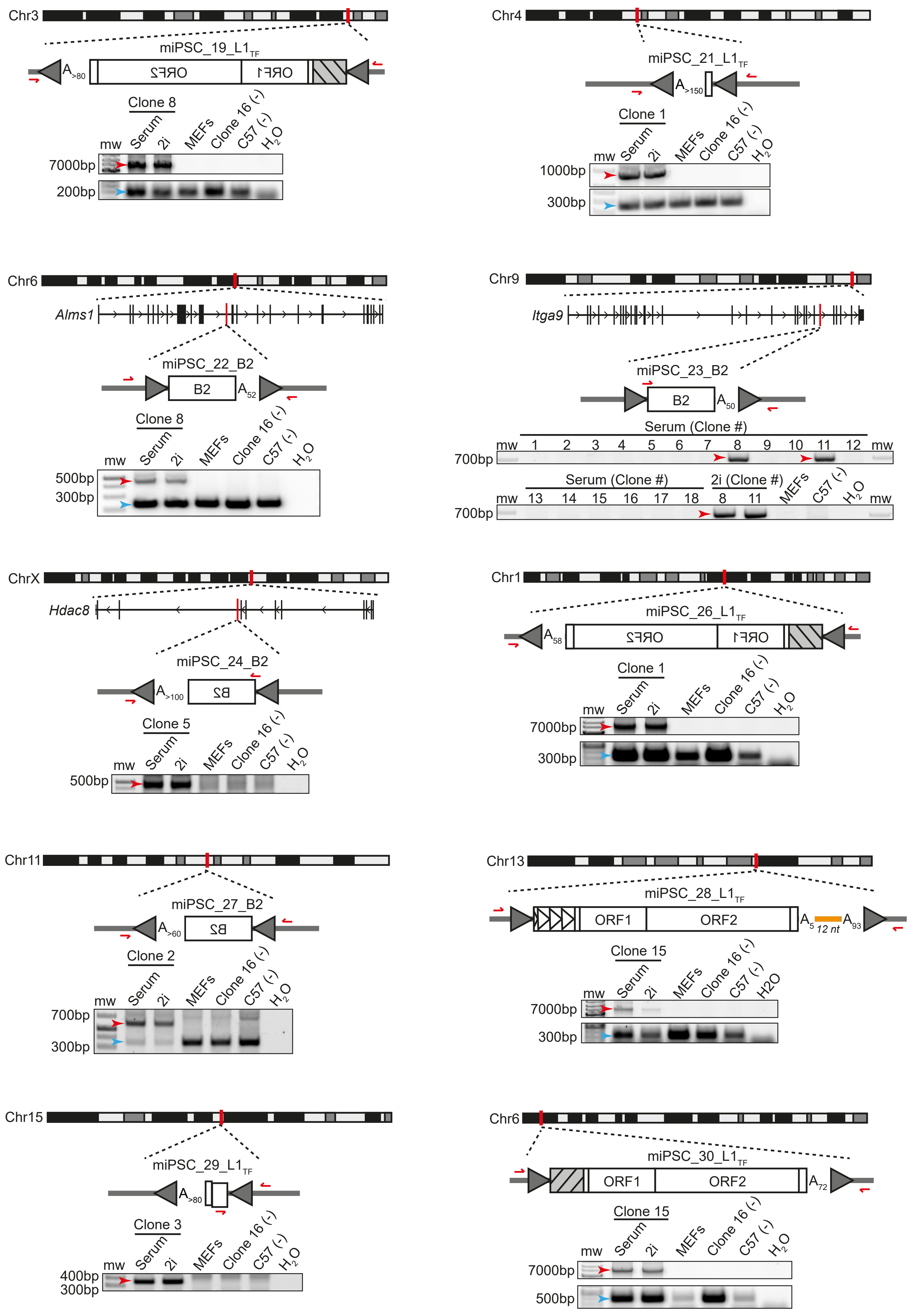

Chr3
miPSC_19_L1TF
A>80
ORF2
ORF1
Clone 8
mw
Serum
2i
MEFs
Clone 16 (-)
C57 (-)
H2O
7000bp
200bp
Chr4
miPSC_21_L1TF
A>150
Clone 1
1000bp
300bp
Chr6
Alms1
miPSC_22_B2
B2
A52
Clone 8
500bp
300bp
Chr9
Itga9
miPSC_23_B2
A50
Serum (Clone #)
1 2 3 4 5 6 7 8 9 10 11 12
13 14 15 16 17 18
2i (Clone #)
8 11
700bp
ChrX
Hdac8
miPSC_24_B2
A>100
Clone 5
Chr1
miPSC_26_L1TF
A58
Clone 1
Chr11
miPSC_27_B2
A>60
Clone 2
700bp
300bp
Chr13
miPSC_28_L1TF
A5
12 nt
A93
Clone 15
H2O
Chr15
miPSC_29_L1TF
Clone 3
400bp
300bp
Chr6
miPSC_30_L1TF
A72
Clone 15
500bp

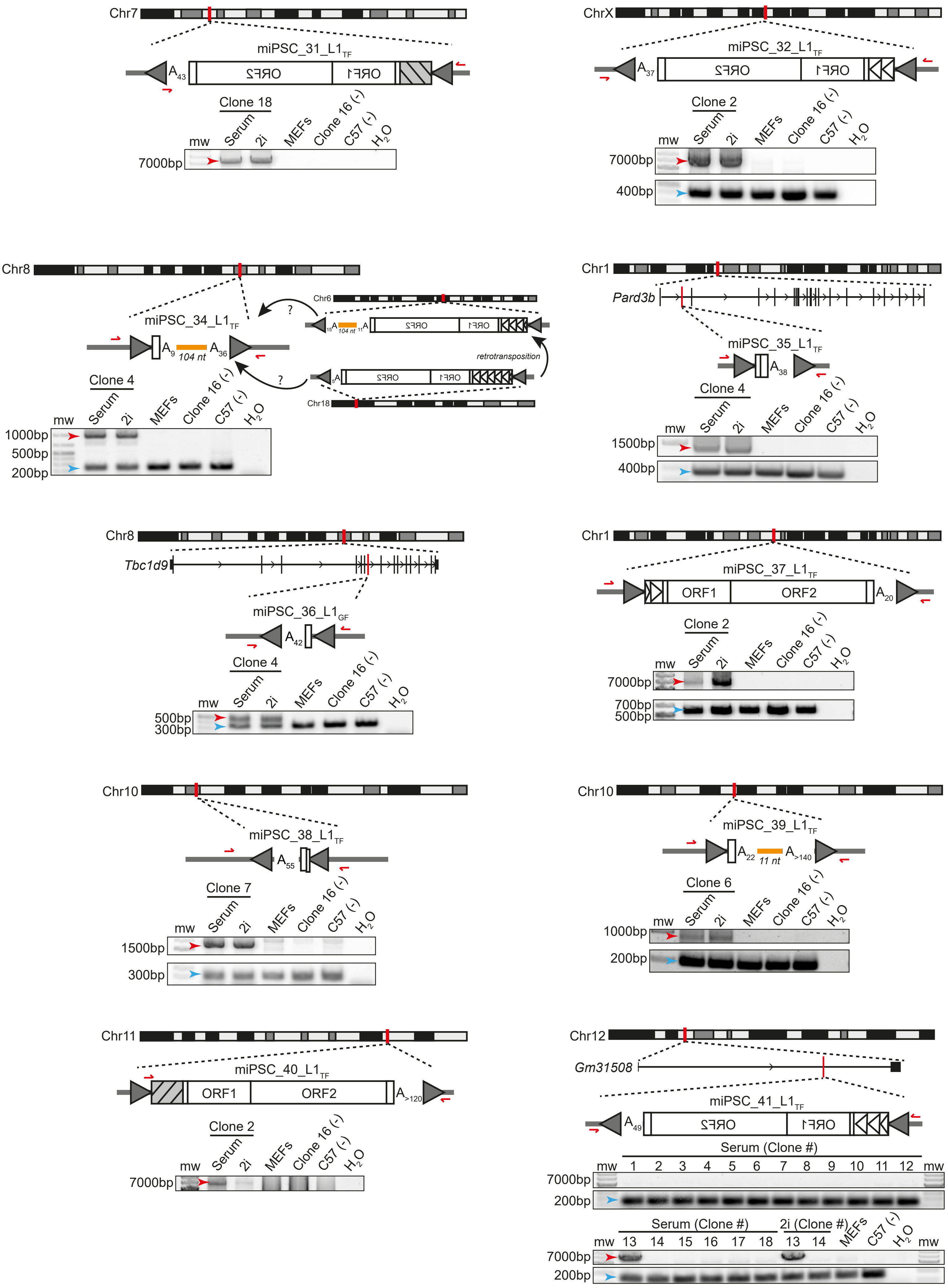
Chr7
miPSC_31_L1TF
A43
Clone 18
mw
Serum
2i
MEFs
Clone 16 (-)
C57 (-)
H2O
7000bp
ChrX
miPSC_32_L1TF
A37
Clone 2
400bp
Chr8
Chr6
miPSC_34_L1TF
A9
104 nt
A36
retrotransposition
Chr18
Clone 4
1000bp
500bp
200bp
Chr1
Pard3b
miPSC_35_L1TF
A38
1500bp
Tbc1d9
miPSC_36_L1GF
A42
300bp
miPSC_37_L1TF
ORF1
ORF2
A20
700bp
Chr10
miPSC_38_L1TF
A55
Clone 7
miPSC_39_L1TF
A22
11 nt
A>140
Clone 6
Chr11
miPSC_40_L1TF
A>120
Chr12
Gm31508
miPSC_41_L1TF
A49
Serum (Clone #)
1 2 3 4 5 6 7 8 9 10 11 12
Serum (Clone #)
13 14 15 16 17 18
2i (Clone #)
13 14

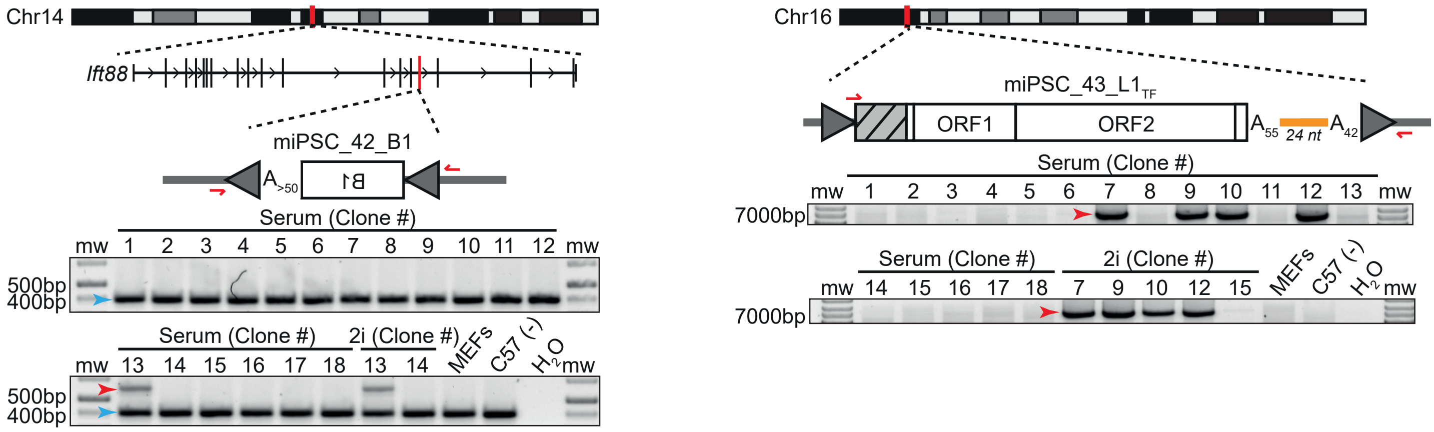

Supplementary Fig. 3. Additional de novo TE insertion validation and characterization. De novo TE insertions found in 26 bulk miPSC lines generated from primary cells or 18 single-cell miPSC clones derived from MEFs, each subjected to Illumina sequencing. For each insertion, the chromosomal location and orientation are shown. L1 and SINE B1 and B2 insertions are represented by white rectangles. L1 5′UTR promoter monomers, if present, are indicated by triangles or, if the number of monomers is unknown, a gray box with black stripes. Poly(A) tracts and their length are indicated (A_n), and target site duplications (TSDs) are depicted as gray arrows. 3′ transductions are shown as orange lines. PCR validation primers are shown as red arrows. Molecular weight (mw) markers are provided at the left of each gel. PCR products in agarose gels used to confirm TE insertions are indicated by red arrows. Empty site (wild-type) amplicons are indicated by blue arrows, where applicable. Source data are provided as a Source Data file.

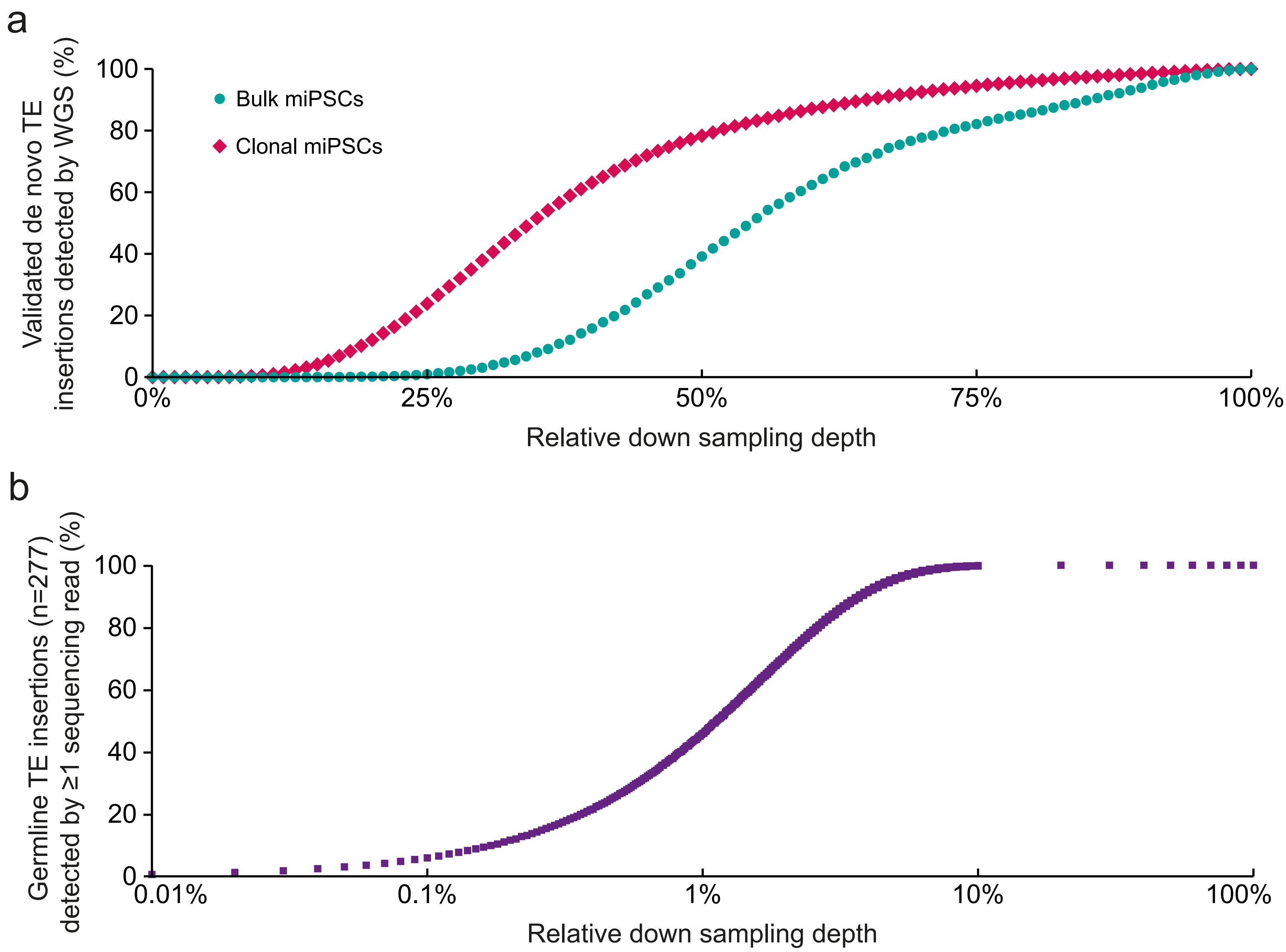

Supplementary Fig. 4. TE detection sensitivities at simulated sequencing depths. a, To assess whether PCR validated de novo TE insertions would have been initially overlooked by lower coverage WGS, we down sampled our ~41× average depth WGS in percentile increments. In order to be called as present, de novo insertions found in the bulk (top) and single-cell (bottom) miPSC experiments required ≥1 WGS read at each of their 5′ and 3′ junctions, and ≥10 WGS reads in total. **b,** To estimate the likelihood of a mosaic TE insertion being overlooked in the parental animal I222e2 MEF population, and called as de novo in one of the associated clonal miPSC lines, we defined a set of 277 heterozygous germline TE insertions found in I222e2 and that were detected by ≥25 WGS or mRC-seq reads at each of their 5′ and 3′ junctions. We then simulated the probability of at least one read being found for an insertion when the reads assigned to that insertion were assigned probabilities to achieve random sampling depths ranging from 0.01% to 100% of the parental MEF bulk sequencing data. Note: at each depth in panels (a) and (b), simulations were repeated 10,000 times.

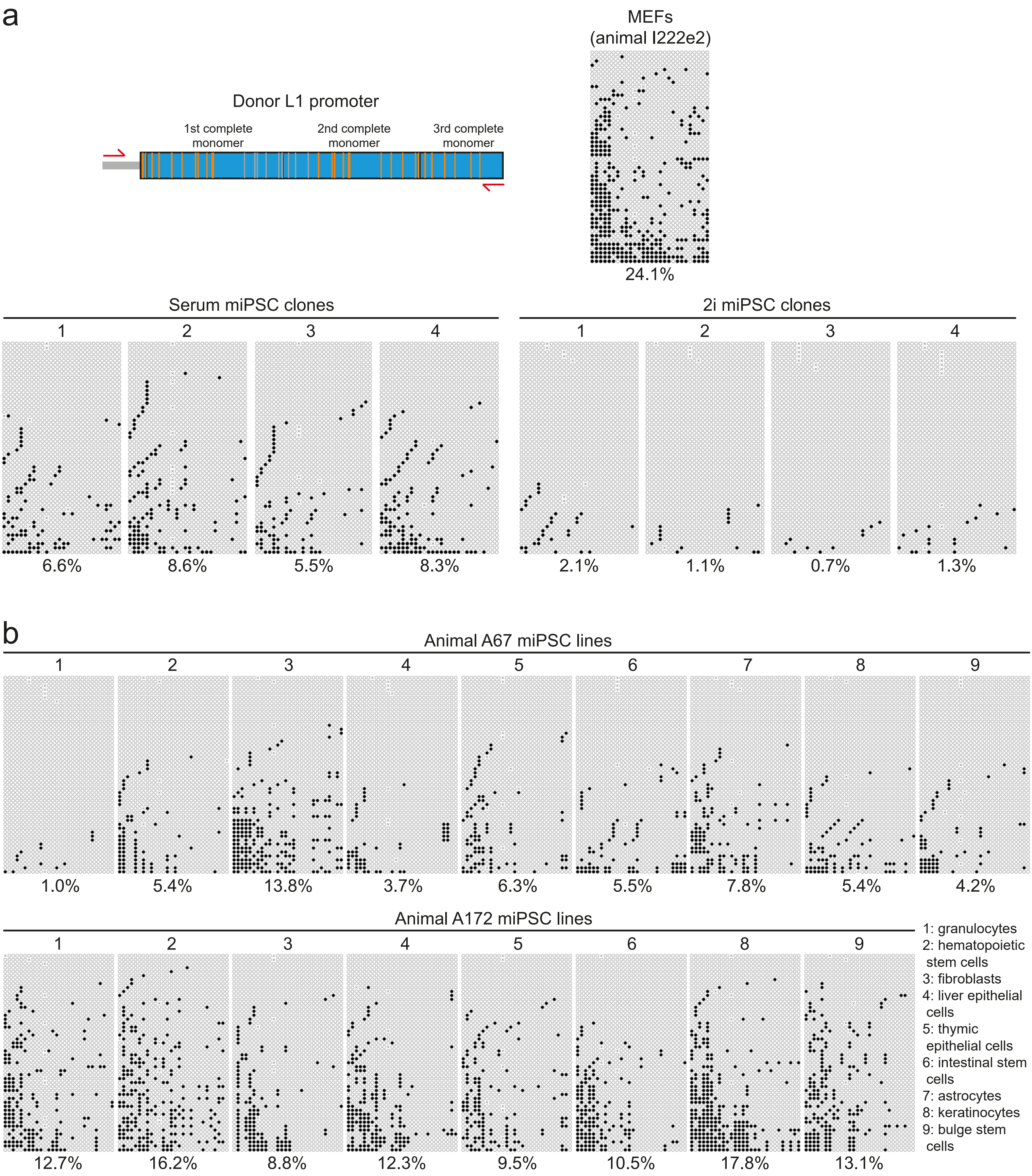

Supplementary Fig. 5. Donor L1 hypomethylation in MEFs and miPSCs. a, top left: Locus-specific methylation analysis design for a donor L1 found to generate insertion miPSC_10_L1 in a MEF-derived single-cell miPSC clone (Clone 1). CpGs located in the first 3 monomers of the donor L1 were assessed. Orange and gray strokes indicate CpGs covered and not covered, respectively, by sequencing the amplicon with 2×300mer Illumina reads. bottom right: Methylation of the donor L1 promoter sequence in four single-cell miPSC clones, including Clone 1, cultured in either serum or 2i conditions, and the parental MEF population. Each cartoon panel corresponds to an amplicon and displays 50 non-identical randomly selected sequences (black circle, methylated CpG; white circle, unmethylated CpG; ×, mutated CpG). The percentage of methylated CpG is indicated underneath each cartoon. **b,** Donor L1 methylation data as per (a) except for bulk miPSC lines derived from two animals (A67 and A172) carrying the polymorphic donor L1.

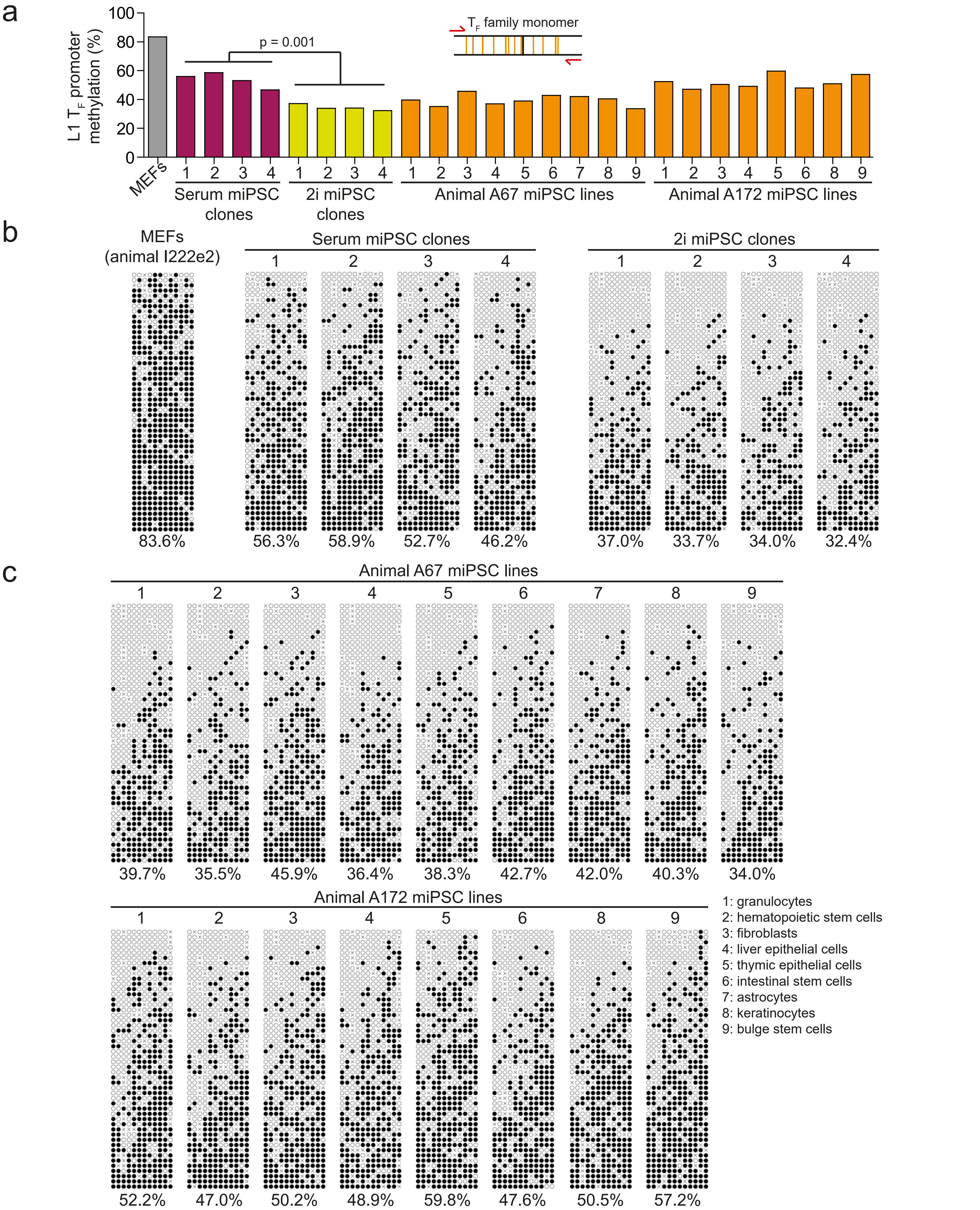

Supplementary Fig. 6. L1 T_F subfamily promoter monomer methylation. a, L1 T_F monomer CpG methylation in MEFs, single-cell miPSC clones, and bulk miPSCs derived from primary cells. top: Assay design and primer locations with respect to L1 T_F monomer structure. Orange strokes indicate CpGs covered by the assay. bottom: Histogram data represent the mean percentage methylation of 50 non-identical bisulfite converted sequences selected at random from each sample. A two-tailed t test (p=0.001) was used to compare serum and 2i culture conditions for single-cell miPSC clones 1-4. **b,** L1 T_F methylation in four single-cell miPSC clones and parental MEFs. Each cartoon panel corresponds to an amplicon and displays 50 non-identical randomly selected sequences (black circle, methylated CpG; white circle, unmethylated CpG; ×, mutated CpG). Methylated CpG percentage is indicated underneath each cartoon. **c,** As per (b) except for bulk miPSCs derived from animals A67 and A172. Note: this assay surveys CpG methylation for T_F monomers genome-wide without retaining their position within individual L1 loci.

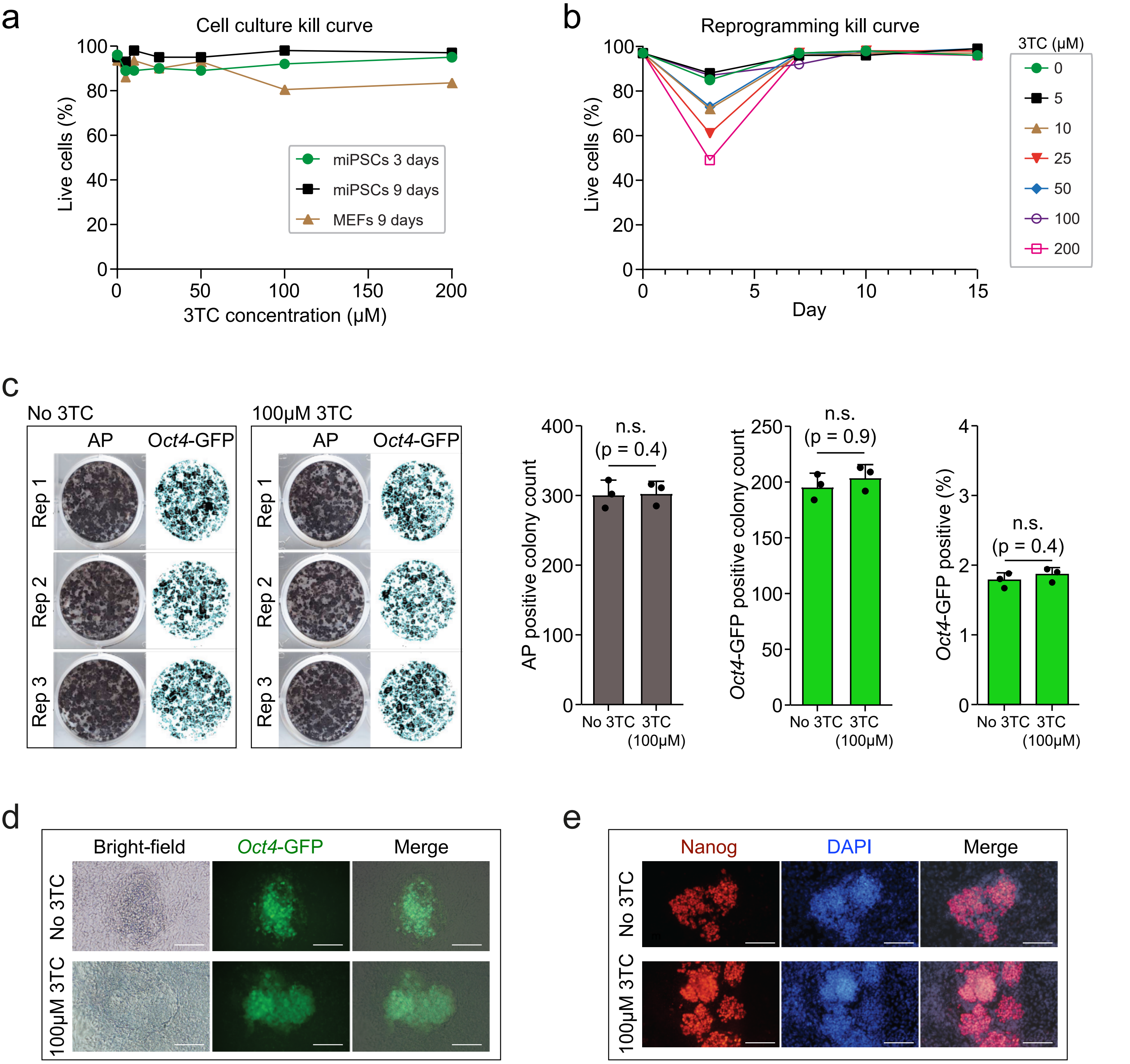

Supplementary Fig. 7. Lamivudine does not impact reprogramming efficiency or miPSC survival. a, Cultured MEF and miPSC viability as a function of lamivudine (3TC) concentration. miPSCs were tested for 3 and 9 days in culture with 3TC, and MEFs tested for 9 days. **b,** Cell viability during MEF reprogramming to miPSCs in the presence of varying concentrations of 3TC, as a function of days since reprogramming was induced by the addition of doxycycline. **c,** Reprogrammed MEFs stained for alkaline phosphatase (AP) and *Oct4*-GFP pluripotency markers 15 days after the addition of doxycycline, in media containing 100µM 3TC or no 3TC. Results for independent biological triplicates (n=3) are shown as microscopy images of stained cultured cells (left) and as histograms (right) representing the mean ± SD observed AP positive colony counts, *Oct4*-GFP positive colony counts, and reprogramming efficiency (percentage *Oct4*-GFP positive cells at day 15 divided by the number of cells seeded at day 0). Differences were not significant (two-tailed t test). **d,** Representative microscopy images of day 15 miPSC colonies obtained when MEFs were reprogrammed in the presence of 100µM 3TC or no 3TC. **e,** Representative immunostaining of Nanog (red), an additional pluripotency marker, and nuclei (DAPI), in day 15 miPSCs. Note: white scale bars in (d) and (e) represent 200µm. Source data are provided as a Source Data file.

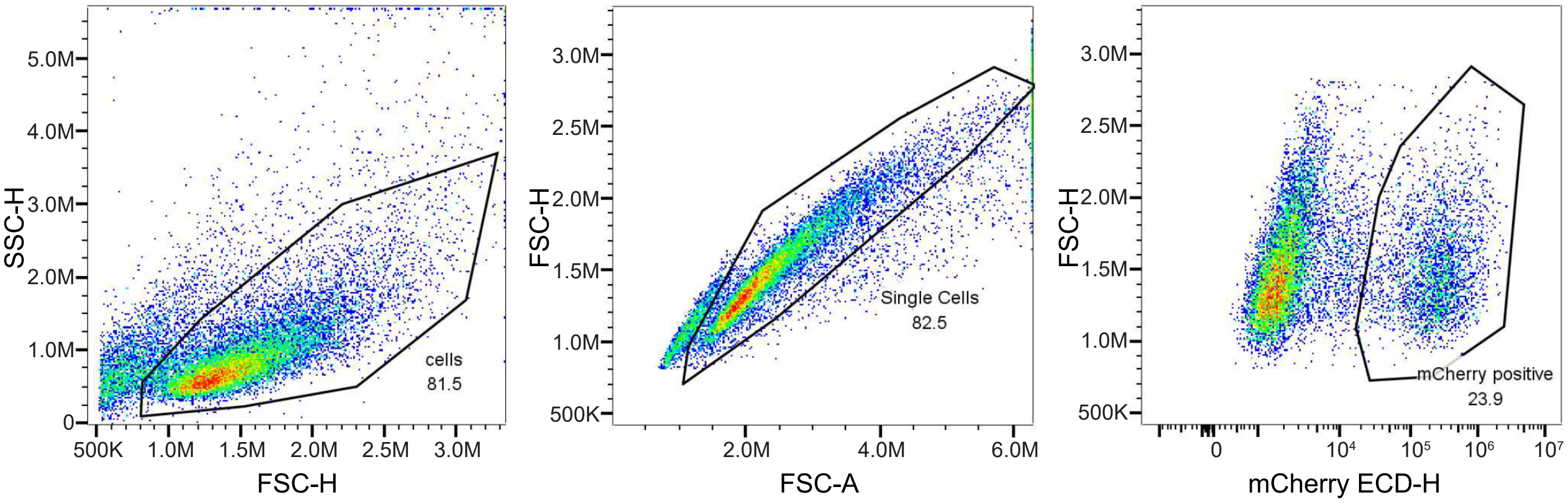

Supplementary Fig. 8. Flow cytometry gating strategy for wild-type L1 T_F mCherry retrotransposition reporter assays conducted in HeLa cells, as shown in Fig. 3b. Cells were gated on an FSC-H (forward scatter - height) versus SSC-H (side scatter - height) plot to exclude debris (left) and then gated on an FSC-A (forward scatter - area) versus FSC-H plot to retain single cells and exclude doublets (middle). mCherry positive cells were detected on the ECD channel (height) versus FSC-H (right).

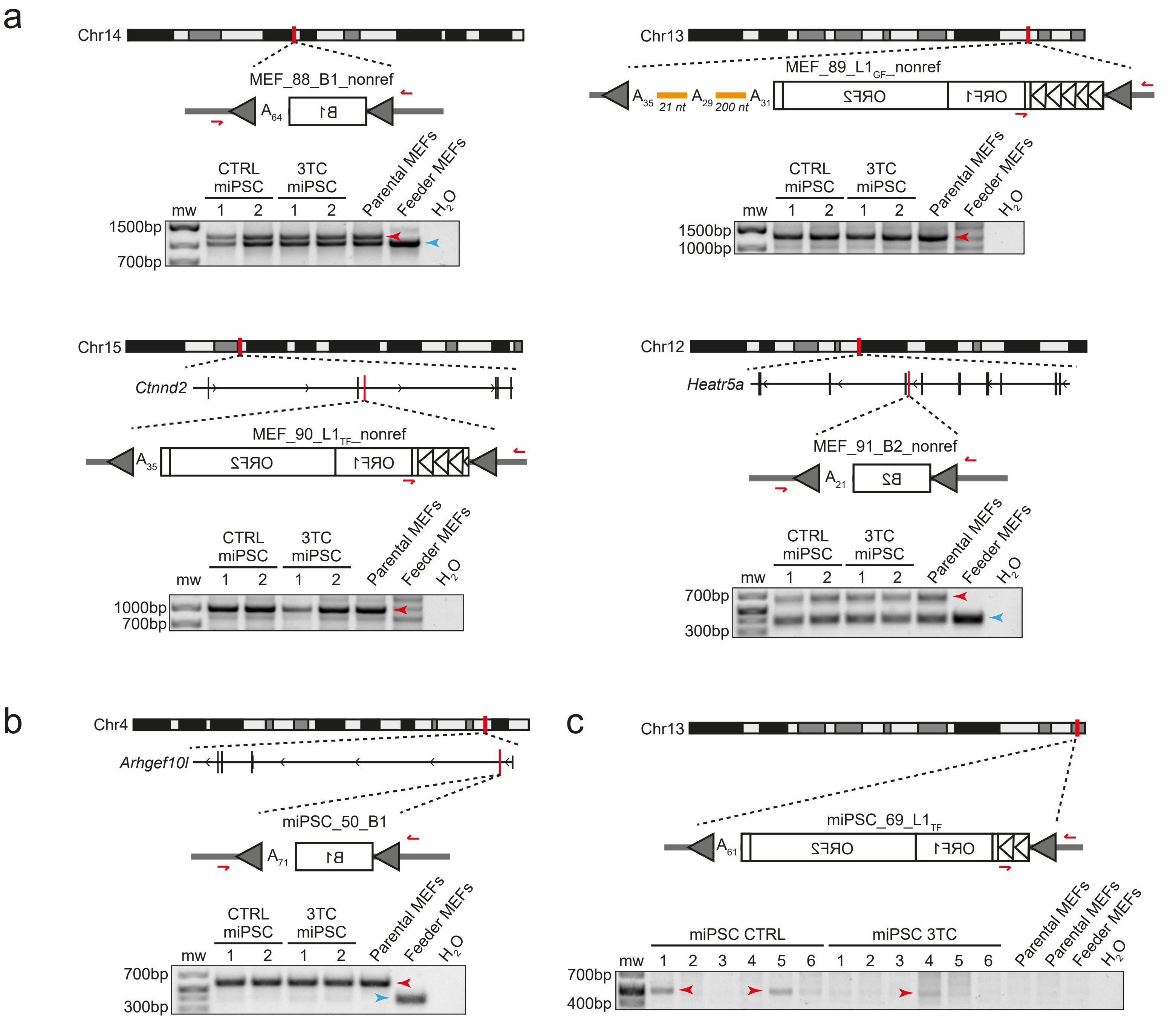

Supplementary Fig. 9. Supporting data for ONT sequencing analysis of miPSCs. a, Non-reference polymorphic TE insertions found by ONT sequencing, used as positive controls for PCR validation experimental designs. **b,** A putative de novo B1 insertion detected in one miPSC line by ONT sequencing and annotated as a false positive based on PCR amplification in the parental MEF template DNA. **c,** A putative de novo L1 T_F insertion detected in one miPSC line by ONT sequencing and amplified in multiple miPSC lines by PCR. Here, DNA was obtained from two parental MEF aliquots, one corresponding to miPSC CTRL/3TC lines 1-3 and one to miPSC CTRL/3TC lines 4-6. Note: in each panel, the insertion chromosomal location and orientation are shown. L1 and SINE B1 and B2 insertions are represented by white rectangles. L1 5′UTR promoter monomers are indicated by triangles. PolyA tracts and their length are indicated (A_n), and target site duplications (TSDs) are depicted as gray arrows. 3′ transductions are shown as orange lines. PCR validation primers are shown as red arrows. Molecular weight (mw) markers are provided at the left of each gel. PCR products in agarose gels used to confirm TE insertions are indicated by red arrows. Empty site (wild-type) amplicons are indicated by blue arrows, where applicable. Source data are provided as a Source Data file.

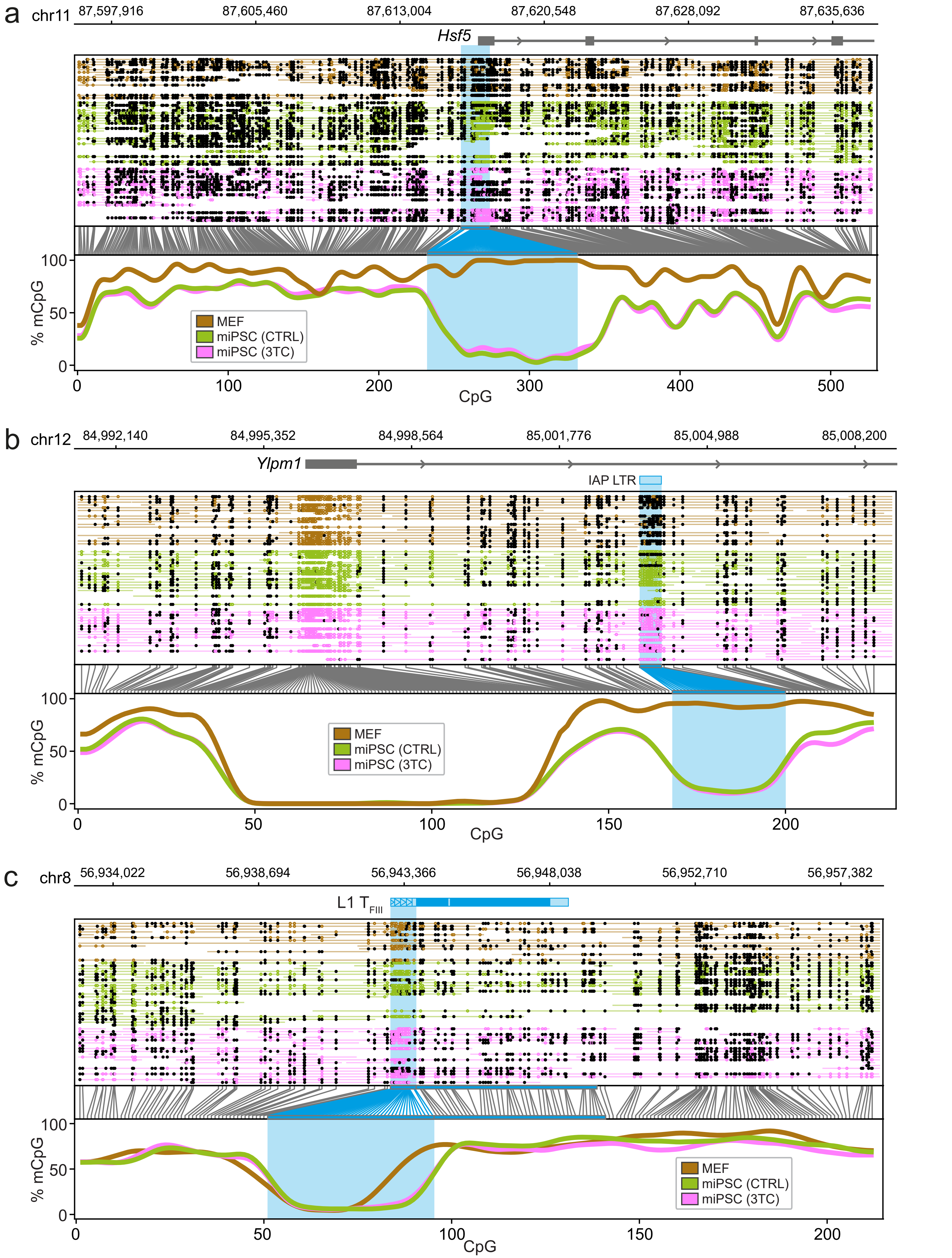

Supplementary Fig. 10. Examples of protein-coding gene and TE methylation, as surveyed by ONT sequencing. Methylation profiles are shown for **a,** the *Hsf5* gene promoter **b,** an IAP LTR intronic to *Ylpm1*, and **c,** an intergenic L1 T_F. For each example, the panels are arranged as per Fig. 4c. ONT data are shown for MEFs (brown) and for control (green) and 3TC-treated (pink) miPSC datasets. miPSC replicates were aggregated for each condition and down-sampled to approximate MEF dataset read depth.
