# Supplementary material for: Retrotransposon instability dominates the acquired mutation landscape of mouse induced pluripotent stem cells
Source: Nat Commun. 2022 Dec 3;13:7470. doi: 10.1038/s41467-022-35180-x (PMC9719517; doi:10.1038/s41467-022-35180-x)
Supplement: Supplementary file 3 — Description of additional Supplementary File [file 41467_2022_35180_MOESM3_ESM.pdf]

Descriptions of additional supplementary files

Supplementary Data 1. WGS and mRC-seq library statistics.

Supplementary Data 2. Genomic variants private to individual bulk or single-cell miPSC lines, and their predicted impacts. Note: Variants (SNVs, INDELs, SVs) were called using Illumina WGS data only.

Supplementary Data 3. TE insertion validation details. Four tabs provide information for de novo insertions detected via (i) Illumina sequencing of bulk miPSCs reprogrammed from various tissues, (ii) Illumina sequencing of single-cell miPSC clones derived from MEFs, (iii) ONT sequencing of bulk miPSCs derived from MEFs and (iv) ONT sequencing to detect L1-mCherry retrotransposition events in cultured HeLa cells. Illumina data were analyzed with TEBreak, while ONT data were analyzed with TLDR. In tabs (i), (ii) and (iii), Column 1 lists a universally unique identifier (UUID) for each insertion. Columns 2-6 show the insertion name used in other figures and tables, the chromosomal location including genomic 5′ and 3′ end positions and whether the insertion is intergenic or intragenic. For tabs (i) and (ii), columns 7-14 provide the 5′ and 3′ end orientations and whether these provisionally indicate a 5′ inversion, the TE family, the 5′ and 3′ end nucleotide positions of each insertion relative to the TE consensus sequence, and the WGS/mRC-seq read counts for the 5′ and 3′ ends. Column 15 displays the TSD sequence and columns 16 and 17 respectively contain the 5′ and 3′ junction consensus sequences constructed from WGS/mRC-seq reads. Columns 18 and 19 contain the name of the samples where the TE insertion was detected at its 5′ and 3′ end, respectively, and the number of reads present for each sample. For tab (iii), columns 7-17 provide the orientation of the insertion, the TE family and subfamily, the 5′ and 3′ end nucleotide positions of each insertion relative to the TE consensus sequence, the length of the insertion, whether a 5′ inversion is present, the total number of ONT reads supporting the insertion, the total number of spanning reads supporting the insertion, the name of the samples where the TE insertion was detected and the number of reads present for each sample, and whether the TE insertion has previously been observed in genomic analysis of mouse strains. Columns 18-20 display the TSD sequence, the consensus sequencing constructed from ONT reads, and any associated TLDR flags. Columns 21-23 indicate TSD sequences and other TPRT hallmarks associated with TE insertions obtained by manual inspection of consensus sequences. For tab (iv), Column 1 lists a universally unique identifier (UUID) for each insertion. Columns 2-4 show the chromosomal location including genomic 5′ and 3′ end positions. Columns 5-13 provide the orientation of the insertion, the TE family (all L1-mCherry), the 5′ and 3′ end nucleotide positions of each insertion relative to the spliced L1-mCherry sequence, the length of the insertion, whether a 5′ inversion is present, the total number of ONT reads supporting the insertion, the total number of spanning reads supporting the insertion, and the name of the HeLa colony carrying the TE insertion along with the corresponding number of reads. Columns 14-16 display the TSD sequence, the

consensus sequencing constructed from ONT reads, and any associated TLDR flags. Columns 17-19 indicate TSD sequences and other TPRT hallmarks obtained by manual inspection of consensus sequences. Finally, in tabs (i), (ii) and (iii), the last 10 columns comprise the validation PCR type, validation PCR primers and capillary sequencing reads for each insertion, and any relevant notes.

Supplementary Data 4. Non-reference TE insertions detected in MEFs. Two tabs provide information for (i) 277 gold standard heterozygous insertions detected via Illumina sequencing and (ii) insertions detected via ONT sequencing. Each tab is arranged approximately as per Supplementary Data 3, except without PCR validation information. All listed insertions were detected in the parental MEFs.

Supplementary Data 5. Reference genome TE methylation values. Tab (i) contains information for TEs and tab (ii) contains information for gene promoters, as defined by the Eukaryotic Promoter Database. Columns are as follows. seg_chrom, seg_start, seg_end: position of the reference TE or gene promoter. seg_name: TE subfamily or gene promoter name. seg_strand: + or - orientation relative to the genome assembly. *_meth_calls: number of methylated CpG calls for each sample. *_unmeth_calls: number of unmethylated CpG calls for each sample. *_no_calls: number of ambiguous CpG calls for each sample (log likelihood ratio between -2.5 and 2.5). *_methfrac: fraction of non-ambiguous calls indicating methylation. *_readcount: number of reads aligned to the element. methfrac_difference: difference between the indicated sample methylation percentages. uncorrected_pvalue: Fisher's exact test two-sided p-value from comparison of methylation/demethylation counts between samples. fdr_corrected_pvalue: the value in "uncorrected_pvalue" Bonferroni corrected for multiple testing. significant: whether the corrected p-value is less than 0.01 and the absolute methylation difference (ΔmC) is >25%. In tab (i), L1_TSS_support indicates whether the 5′ end of a spliced EST or mRNA obtained from the UCSC Genome Browser coincides with the listed L1. Note: full-length L1 methylation values were calculated for the given 5′UTR coordinates.
