# Supplementary material for: Retrotransposon instability dominates the acquired mutation landscape of mouse induced pluripotent stem cells
Source: Nat Commun. 2022 Dec 3;13:7470. doi: 10.1038/s41467-022-35180-x (PMC9719517; doi:10.1038/s41467-022-35180-x)
Supplement: Supplementary file 9 — Reporting Summary [file 41467_2022_35180_MOESM9_ESM.pdf]

nature portfolio

Corresponding author(s): Sandra R Richardson, Jose M Polo, Geoffrey J Faulkner

Last updated by author(s): Nov 7, 2022

Reporting Summary

Nature Portfolio wishes to improve the reproducibility of the work that we publish. This form provides structure for consistency and transparency in reporting. For further information on Nature Portfolio policies, see our Editorial Policies and the Editorial Policy Checklist.

Statistics

For all statistical analyses, confirm that the following items are present in the figure legend, table legend, main text, or Methods section.

n/a	Confirmed	
☐	☒	The exact sample size (*n*) for each experimental group/condition, given as a discrete number and unit of measurement
☐	☒	A statement on whether measurements were taken from distinct samples or whether the same sample was measured repeatedly
☐	☒	The statistical test(s) used AND whether they are one- or two-sided<br>*Only common tests should be described solely by name; describe more complex techniques in the Methods section.*
☒	☐	A description of all covariates tested
☐	☒	A description of any assumptions or corrections, such as tests of normality and adjustment for multiple comparisons
☐	☒	A full description of the statistical parameters including central tendency (e.g. means) or other basic estimates (e.g. regression coefficient) AND variation (e.g. standard deviation) or associated estimates of uncertainty (e.g. confidence intervals)
☐	☒	For null hypothesis testing, the test statistic (e.g. *F*, *t*, *r*) with confidence intervals, effect sizes, degrees of freedom and *P* value noted<br>*Give P values as exact values whenever suitable.*
☒	☐	For Bayesian analysis, information on the choice of priors and Markov chain Monte Carlo settings
☒	☐	For hierarchical and complex designs, identification of the appropriate level for tests and full reporting of outcomes
☒	☐	Estimates of effect sizes (e.g. Cohen's *d*, Pearson's *r*), indicating how they were calculated

Our web collection on statistics for biologists contains articles on many of the points above.

Software and code

Policy information about availability of computer code

Data collection	Software installed on Illumina and Oxford Nanopore Technologies sequencing platforms was used for data collection.
Data analysis	This study used the following software, as described in the Methods:<br><br>TEBreak, version 1.1, https://github.com/adamewing/tebreak<br>TLDR, version 1.2.2, https://github.com/adamewing/TLDR<br>Methylartist, version 1.2.4, https://github.com/adamewing/methylartist<br>QUMA, version 1.1.16 http://quma.cdb.riken.jp/<br>bwa-mem, version 0.7.12, https://github.com/lh3/bwa<br>Picard MarkDuplicates, version, 1.128, https://gatk.broadinstitute.org/hc/en-us<br>Picard DownsampleSam, version 2.27.4, https://gatk.broadinstitute.org/hc/en-us<br>GATK IndelRealigner, version 3.7, https://gatk.broadinstitute.org/hc/en-us<br>GATK HaplotypeCaller, version 3.7, https://gatk.broadinstitute.org/hc/en-us<br>freebayes, version 1.3.1, https://github.com/freebayes/freebayes<br>Delly2, version 0.7.9, https://github.com/dellytools/delly<br>GRIDSS, version 2.0.0, https://github.com/PapenfussLab/gridss<br>SnpEff, version 4.3T, http://pcingola.github.io/SnpEff/<br>minimap2, version 2.20, https://github.com/lh3/minimap2<br>SAMtools, version 1.12, https://github.com/samtools/samtools<br>nanopolish, version 0.13.2, https://github.com/jts/nanopolish<br>guppy, version 5.0.13, https://community.nanoporetech.com/downloads/<br>Serial Cloner, version 2.6, http://serialbasics.free.fr/Serial_Cloner.html

UCSC Genome Browser BLAT, version 438, https://genome.ucsc.edu/cgi-bin/hgBlat
Repbase CENSOR, version 4.2.22, https://www.girinst.org/censor/
FlowJo, version 10.8.1, https://www.flowjo.com/solutions/flowjo/downloads/
CytExpert, version 2.5, https://www.beckman.com.au/flow-cytometry/research-flow-cytometers/cytoflex/
SciPy, version 1.4.1, https://scipy.org/
Seaborn, version 0.9, https://seaborn.pydata.org/

For manuscripts utilizing custom algorithms or software that are central to the research but not yet described in published literature, software must be made available to editors and reviewers. We strongly encourage code deposition in a community repository (e.g. GitHub). See the Nature Portfolio guidelines for submitting code & software for further information.

Data

Policy information about availability of data

All manuscripts must include a data availability statement. This statement should provide the following information, where applicable:

- Accession codes, unique identifiers, or web links for publicly available datasets
- A description of any restrictions on data availability
- For clinical datasets or third party data, please ensure that the statement adheres to our policy

All Oxford Nanopore Technologies and Illumina sequencing data generated by this study were deposited in the European Nucleotide Archive (ENA) under project PRJEB20569 (https://www.ebi.ac.uk/ena/browser/view/PRJEB20569). Gene promoter coordinates were obtained from the Eukaryotic Promoter Database (https://epd.epfl.ch/) and reference TE coordinates were defined by the UCSC Genome Browser RepeatMasker track (https://hgdownload.soe.ucsc.edu/goldenPath/mm10/)

Field-specific reporting

Please select the one below that is the best fit for your research. If you are not sure, read the appropriate sections before making your selection.

☒ Life sciences ☐ Behavioural & social sciences ☐ Ecological, evolutionary & environmental sciences

For a reference copy of the document with all sections, see nature.com/documents/nr-reporting-summary-flat.pdf

Life sciences study design

All studies must disclose on these points even when the disclosure is negative.

Sample size	No statistical method was used to predetermine sample size. Experiments were performed to at least obtain biological triplicate data. 9 primary cell types were obtained from 3 animals and reprogrammed to miPSCs (n=3 from each cell type apart from astrocytes (n=2)). Mouse embryonic fibroblasts were used to generate an additional cohort of 18 single-cell miPSC clones, and 12 bulk-reprogrammed miPSC populations. These sample sizes were chosen to, firstly, obtain biological triplicates and, secondly, to generate the largest possible dataset given practical limitations.
Data exclusions	No data were excluded from the analyses. Insufficient astrocyte-derived miPSCs were obtained from animal A172 to perform a genomic analysis for that one sample.
Replication	Genomic analyses involved at least independent triplicates of miPSC lines as outlined in the Sample Size section. All de novo retrotransposon insertions detected by whole genome sequencing were assayed by PCR and capillary sequencing, and considered validated if amplified as an on-target product at least one. Retrotransposition reporter assays were conducted in triplicate and generated consistent results across replicates.
Randomization	The experiments were not randomized. The study did not involve randomized samples.
Blinding	The investigators were not blinded to sample identity during sample selection, collection or analysis. No analysis involved subjective observations, and the same analysis approach was applied to samples in different groups, for example where lamivudine-treated miPSCs were compared to control miPSCs.

Reporting for specific materials, systems and methods

We require information from authors about some types of materials, experimental systems and methods used in many studies. Here, indicate whether each material, system or method listed is relevant to your study. If you are not sure if a list item applies to your research, read the appropriate section before selecting a response.

Materials & experimental systems

n/a	Involved in the study
	☒ Antibodies
	☒ Eukaryotic cell lines
☒	Palaeontology and archaeology
	☒ Animals and other organisms
☒	Human research participants
☒	Clinical data
☒	Dual use research of concern

Methods

n/a	Involved in the study
☒	ChIP-seq
	☒ Flow cytometry
☒	MRI-based neuroimaging

Antibodies

Antibodies used

The following antibodies were used to purify primary cell types:

CD5 (BD Biosciences, Cat#: 553020) https://www.bdbiosciences.com/content/dam/bdb/products/global/reagents/flow-cytometry-reagents/research-reagents/single-color-antibodies-ruo/553020_base/pdf/553020.pdf

B220 (BD Biosciences, Cat#: 557669) https://www.bdbiosciences.com/content/dam/bdb/products/global/reagents/flow-cytometry-reagents/research-reagents/single-color-antibodies-ruo/557669_base/pdf/557669.pdf

TER-119 (BD Biosciences, Cat#: 557915) https://www.bdbiosciences.com/content/dam/bdb/products/global/reagents/flow-cytometry-reagents/research-reagents/single-color-antibodies-ruo/557915_base/pdf/557915.pdf

Sca-1 (Biolegend, Cat#: 122519) https://d1spbj2x7qk4bg.cloudfront.net/en-us/products/pacific-blue-anti-mouse-ly-6a-e-sca-1-antibody-3901?pdf=true&displayInline=true&leftRightMargin=15&topBottomMargin=15&filename=Pacific%20Blue%E2%84%A2%20anti-mouse%20Ly-6A/E%20(Sca-1)%20Antibody.pdf&v=20170220122420

cKit (BD Biosciences, Cat#: 553356) https://www.bdbiosciences.com/content/dam/bdb/products/global/reagents/flow-cytometry-reagents/research-reagents/single-color-antibodies-ruo/553356_base/pdf/553356.pdf

SSEA1 (Thermo Fisher Scientific, Cat#: 13-8813-82) https://www.thermofisher.com/order/genome-database/dataSheetPdf?producttype=antibody&productsubtype=antibody_primary&productId=13-8813-82

Gr-1 (Biolegend, Cat#: 108423) https://d1spbj2x7qk4bg.cloudfront.net/en-us/products/apc-cyanine7-anti-mouse-ly-6gly-6c-gr-1-antibody-3935?pdf=true&displayInline=true&leftRightMargin=15&topBottomMargin=15&filename=APC/Cyanine7%20anti-mouse%20Ly-6G/Ly-6C%20(Gr-1)%20Antibody.pdf&v=20220928092644

Mac1 (Biolegend, Cat#: 101207) https://d1spbj2x7qk4bg.cloudfront.net/en-us/products/pe-anti-mouse-human-cd11b-antibody-349?pdf=true&displayInline=true&leftRightMargin=15&topBottomMargin=15&filename=PE%20anti-mouse/human%20CD11b%20Antibody.pdf&v=20220831123135

Streptavidin (BD Biosciences, Cat#: 557598) https://www.bdbiosciences.com/content/dam/bdb/products/global/reagents/flow-cytometry-reagents/research-reagents/single-color-antibodies-ruo/557598_base/pdf/557598.pdf

CD31 (Thermo Fisher Scientific, Cat#: 11-0311-81) https://www.thermofisher.com/order/genome-database/dataSheetPdf?producttype=antibody&productsubtype=antibody_primary&productId=11-0311-81

CD45 (Thermo Fisher Scientific, Cat#: 11-0451-81) https://www.thermofisher.com/order/genome-database/dataSheetPdf?producttype=antibody&productsubtype=antibody_primary&productId=11-0451-81

Thy-1.2 (Thermo Fisher Scientific, Cat#: 17-0902-81) https://www.thermofisher.com/order/genome-database/dataSheetPdf?producttype=antibody&productsubtype=antibody_primary&productId=17-0902-81

EpCAM (Thermo Fisher Scientific, Cat#: 48-5791-82) https://www.thermofisher.com/order/genome-database/dataSheetPdf?producttype=antibody&productsubtype=antibody_primary&productId=48-5791-82

TER-119 (BD Biosciences, Cat#: 560509) https://www.bdbiosciences.com/content/dam/bdb/products/global/reagents/flow-cytometry-reagents/research-reagents/single-color-antibodies-ruo/560509_base/pdf/560509.pdf

MHC Class II (Biolegend, Cat#: 107620) https://d1spbj2x7qk4bg.cloudfront.net/en-us/products/pacific-blue-anti-mouse-i-a-i-e-antibody-3136?pdf=true&displayInline=true&leftRightMargin=15&topBottomMargin=15&filename=Pacific%20Blue%E2%84%A2%20anti-mouse%20I-A/I-E%20Antibody.pdf&v=20220914123035

CD24 (Thermo Fisher Scientific, Cat#: 25-0242-82) https://www.thermofisher.com/order/genome-database/dataSheetPdf?producttype=antibody&productsubtype=antibody_primary&productId=25-0242-82

EphrinB2 (BD Biosciences, Cat#: 743763) https://www.bdbiosciences.com/content/bdb/paths/generate-tds-document.ca.743763.pdf

Alexa Fluor 555 polyclonal (Thermo Fisher Scientific, Cat#: A-31570) https://www.thermofisher.com/order/genome-database/dataSheetPdf?producttype=antibody&productsubtype=antibody_secondary&productId=A-31570

Glast1 (Miltenyi Biotec, Cat#: 130-098-803) https://www.miltenyibiotec.com/upload/assets/dataSheet_p5032_eng_GBR.pdf

CD133 (Thermo Fisher Scientific, Cat#: 12-1331-80) https://www.thermofisher.com/order/genome-database/dataSheetPdf?producttype=antibody&productsubtype=antibody_primary&productId=12-1331-80

CD45 (BD Biosciences, Cat#: 552848) https://www.bdbiosciences.com/content/dam/bdb/products/global/reagents/flow-cytometry-reagents/research-reagents/single-color-antibodies-ruo/552848_base/pdf/552848.pdf

CD31 (Thermo Fisher Scientific, Cat#: 25-0311-82) https://www.thermofisher.com/order/genome-database/dataSheetPdf?producttype=antibody&productsubtype=antibody_primary&productId=25-0311-82

GoH3 (Abcam, Cat#: ab95703) https://www.abcam.com/pe-integrin-alpha-6-antibody-goh3-ab95703.pdf

CD104 (Biolegend, Cat#: 123605) https://d1spbj2x7qk4bg.cloudfront.net/en-us/products/fitc-anti-mouse-cd104-antibody-4491?pdf=true&displayInline=true&leftRightMargin=15&topBottomMargin=15&filename=FITC%20anti-mouse%20CD104%20Antibody.pdf&v=20220121043308

CD34 (Thermo Fisher Scientific, Cat#: 13-0341-85) https://www.thermofisher.com/order/genome-database/dataSheetPdf?producttype=antibody&productsubtype=antibody_primary&productId=13-0341-85

CD45 (BD Biosciences, Cat#: 557659) https://www.bdbiosciences.com/content/dam/bdb/products/global/reagents/flow-cytometry-reagents/research-reagents/single-color-antibodies-ruo/557659_base/pdf/557659.pdf

EpCAM (Biolegend, Cat#: 118214) https://d1spbj2x7qk4bg.cloudfront.net/en-us/products/apc-anti-mouse-cd326-ep-cam-antibody-4974?pdf=true&displayInline=true&leftRightMargin=15&topBottomMargin=15&filename=APC%20anti-mouse%20CD326%20(Ep-CAM)%20Antibody.pdf&v=20220913063022

CD45 (BD Biosciences, Cat#: 563891) https://www.bdbiosciences.com/content/dam/bdb/products/global/reagents/flow-cytometry-reagents/research-reagents/single-color-antibodies-ruo/563891_base/pdf/563891.pdf

CD31 (BD Biosciences, Cat#: 563089) https://www.bdbiosciences.com/content/dam/bdb/products/global/reagents/flow-cytometry-reagents/research-reagents/single-color-antibodies-ruo/563089_base/pdf/563089.pdf

APC-Streptavidin (Biolegend, Cat#: 405207) https://d1spbj2x7qk4bg.cloudfront.net/en-us/products/apc-streptavidin-1470?pdf=true&displayInline=true&leftRightMargin=15&topBottomMargin=15&filename=APC%20Streptavidin.pdf&v=20220609105935

Validation

Validation information for each of these antibodies is available from the manufacturer, and via the links provided alongside the above list of antibodies used.

Eukaryotic cell lines

Policy information about cell lines

Cell line source(s)	HeLa-JVM, a HeLa sub line, was obtained from the laboratory of John V. Moran.
Authentication	None of the cell lines were authenticated.
Mycoplasma contamination	Cells were tested for mycoplasma contamination and returned a negative result.
Commonly misidentified lines (See ICLAC register)	No commonly misidentified cell lines were used in the study.

Animals and other organisms

Policy information about studies involving animals; ARRIVE guidelines recommended for reporting animal research

Laboratory animals	Induced pluripotent stem cells were generated from adult (animal A67: female, 57 days; animal A82: female, 50 days; animal A172 female, 39 days) and embryonic (E13.5) Oct4GFP-OKSM-M2rtTA doxycycline inducible reprogrammable mice on a B6/129S4 background (Stadtfeld et al., 2010) as described in the Methods.
Wild animals	The study did not involve wild animals.
Field-collected samples	The study did not involve samples collected from the field.

Ethics oversight	All animal experimentation was performed under the auspices and approval of the Monash University Animal Research Platform Animal Ethics Committee (Approval Numbers MARP-2011-172-Polo, MARP-2011-171-BC-Polo, MARP-2017-151-BC-Polo, and ERM# 21634).

Note that full information on the approval of the study protocol must also be provided in the manuscript.

Flow Cytometry

Plots

Confirm that:

- [x] The axis labels state the marker and fluorochrome used (e.g. CD4-FITC).
- [x] The axis scales are clearly visible. Include numbers along axes only for bottom left plot of group (a 'group' is an analysis of identical markers).
- [x] All plots are contour plots with outliers or pseudocolor plots.
- [x] A numerical value for number of cells or percentage (with statistics) is provided.

Methodology

Sample preparation	Transfected HeLa cells and untransfected control HeLa cells grown in 6-well tissue culture dishes were trypsinised, pelleted, and resuspended in 300 ul DPBS.
Instrument	CytoFLEX flow cytometer (Beckman Coulter)
Software	Data were analysed using CytExpert software (Beckman Coulter).
Cell population abundance	The starting cell population was a homogeneous HeLa cell culture.
Gating strategy	The cells were gated on a FSC-H vs SSC-H dot-plot with debris excluded. Single cells were gated on a FSC-A vs FSC-H plot. mCherry positive cells were gated on an FSC-H vs. ECD-H plot. Cutoffs for mCherry positive and negative cells were determined based on FSC-H vs ECD-H plots for untransfected negative control cells and pCAG-mCherry transfected positive control cells.

- [x] Tick this box to confirm that a figure exemplifying the gating strategy is provided in the Supplementary Information.
